# Progression of arterial calcifications: what, where, and in whom?

**DOI:** 10.1007/s00330-023-10566-7

**Published:** 2024-01-15

**Authors:** Janine E. van der Toorn, Meike W. Vernooij, M. Arfan Ikram, Maryam Kavousi, Daniel Bos

**Affiliations:** 1 Department of Epidemiology, Erasmus MC, University Medical Center, Rotterdam, The Netherlands; 2 Department of Radiology and Nuclear Medicine, Erasmus MC, University Medical Center, Rotterdam, The Netherlands; 3 Department of Cardiovascular Sciences, KU Leuven, Louvain, Belgium

**Keywords:** Vascular calcification, Multidetector computed tomography, Cardiovascular diseases, Epidemiology

## Abstract

**Objectives:**

There is a lack of information on the development of arteriosclerosis over time. This study aims to assess long-term sex-specific changes in arterial calcifications in five arteries, and the influence of cardiovascular risk factors hereon.

**Methods:**

From a population-based cohort, 807 participants (mean baseline age, 65.8; SD, 4.2) underwent a non-contrast computed tomography (CT) examination between 2003 and 2006, and after a median follow-up of 14 years. We assessed incidences and changes in volumes of coronary artery calcification (CAC), aortic arch calcification (AAC), extracranial (ECAC) and intracranial carotid artery calcification (ICAC), and vertebrobasilar artery calcification (VBAC). We investigated the simultaneous presence of severe progression (upper quartile of percentual change volumes). Associations of cardiovascular risk factors with changes in calcification volumes were assessed using multivariate linear regression models.

**Results:**

The difference in AAC was most substantial; the median volume (mm^3^) increased from of 129 to 916 in men and from 93 to 839 in women. For VBAC, no change in volumes was observed though more than a quarter of participants without baseline VBAC developed VBAC during follow-up. Severe progression was most often observed in only one artery at the same time. Hypertension was most consistently associated with increase in calcifications. Associations of diabetes, hypercholesterolemia, and smoking with changes in calcifications varied across arteries and sex.

**Conclusions:**

We found a considerable incidence and increase in volumes of calcifications in different arteries, over a 14-year time interval. Cardiovascular risk factors were associated with increase of calcifications with sex-specific differential effects across arteries.

**Clinical relevance statement:**

There is a considerable incidence and increase in volumes of calcifications in different arteries, over a 14-year time interval. Cardiovascular risk factors are associated with increase of calcifications with sex-specific differential effects across arteries; thus, assessing changes in only one artery may thus not provide a good reflection of the systemic development of arteriosclerosis.

**Key Points:**

*• Assessing change in arterial calcification in only one artery does not reflect the systemic development of arterial calcification.*

*• Cardiovascular risk factors are associated with progression of arterial calcifications.*

*• Progression of arterial calcification is sex and artery-specific.*

**Graphical Abstract:**

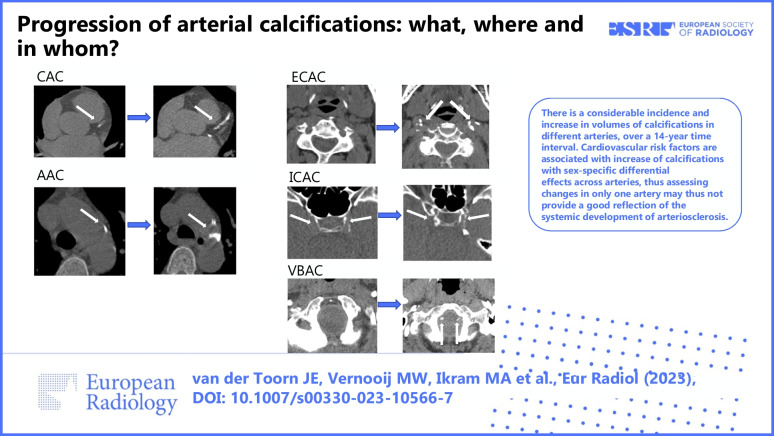

**Supplementary Information:**

The online version contains supplementary material available at 10.1007/s00330-023-10566-7.

## Introduction

Cardiovascular disease events account for one-third of all deaths worldwide, and are mainly attributed to arteriosclerosis [1, 2]. Evidence on the development of arteriosclerosis suggests that it is a highly dynamic process [3, 4], influenced by many endo- and exogenous factors [5]. In-depth research in a real-life setting into the course of arteriosclerosis, and the influence of cardiovascular risk factors hereon, will lead to better understanding the etiology of vascular disease, which in turn could aid optimization of personalized preventive strategies.

There is a lack of information on artery-specific differences in the course of arteriosclerosis. The correlation of arteriosclerosis across different arteries is only weak to moderate [6]. This could be a consequence of artery-specific differences in underlying cardiovascular risk factors, anatomy [7], genetic susceptibility [8], and their interactions. As such, the development of arteriosclerosis might differ per artery, and individuals may benefit from specific preventive action. Particularly on a population-based level, it is largely unknown how arteriosclerosis develops over a long period of time. Only a few population-based studies have focused on long-term changes of arteriosclerosis [9, 10]. However, none have provided head-to-head comparisons of arteriosclerotic changes in multiple major vessel beds covering a timespan of more than a decade. Moreover, cardiovascular disease manifestations differ between men and women [11, 12], but the underlying causes of these differences are incompletely understood. Determining long-term changes in arteriosclerosis and assessment of its cardiovascular risk factors, in men and women separately, would contribute to a more comprehensive understanding of the pathophysiology of arteriosclerosis.

Within a population-based cohort, we determined the sex-specific changes in arterial calcification, as proxy of arteriosclerosis, in the coronary arteries, aortic arch, extracranial carotid arteries, intracranial carotid arteries, and the vertebrobasilar arteries covering a 14-year time interval. We further investigated the sex-specific association of cardiovascular risk factors with changes in arterial calcifications in the different arteries.

## Materials and methods

### Setting and study population

This study is embedded within the Rotterdam Study, a large prospective population-based cohort aimed at investigating determinants and occurrence of chronic diseases. The design has been described in detail previously [13]. Between 2003 and 2006, a random sample of participants visiting the research center [13] were invited to undergo a multidetector computed tomography (CT) scan to quantify arterial calcification, as a proxy for arteriosclerosis, in the following arteries: coronary arteries, aortic arch, extracranial carotid arteries, intracranial carotid arteries, and vertebrobasilar arteries. In total, 2524 participants were scanned (response rate, 78%). Between 2018 and 2020, participants who were still participating in the Rotterdam Study (*n* = 1599) were invited for a second CT examination. During this second examination, a dual source CT scan was used. In total, 951 participants underwent a second examination (response rate, 59.5%). Out of 951 participants with two CT examinations, 144 participants were excluded because quantification of calcification at baseline or follow-up was not possible due to the presence of a coronary stent (*n* = 83), image artefacts (*n* = 33), suboptimal field of view (*n* = 26), or surgical interventions (*n* = 2), leaving 807 participants for current analysis. A STROBE flowchart is provided in the supplementary file (Supplemental Fig. I).

### Assessment of calcification

At baseline, non-contrast CT images were obtained using 16-slice or 64-slice multidetector CT scanners (Somatom Sensation 16 or 64; Siemens). During the follow-up examination, non-contrast CT images were obtained using a 128-slice dual-source CT scanner (Somatom Drive, Siemens). At baseline and follow-up, two scans were performed: a cardiac and a carotid scan [14]. On these scans, calcifications were assessed in the coronary arteries, aortic arch, extracranial, and intracranial carotid arteries, and the vertebrobasilar arteries. Further information on the scan protocol is provided in the supplementary file. Detailed description on the evaluation methods is provided elsewhere [14–18]. Intra-rater and inter-rater reliabilities for the scoring methods have previously been reported to be very good–excellent [17, 19, 20].

### Assessment of cardiovascular risk factors

Cardiovascular risk factors were assessed at the time of the first CT scan (baseline). Information on cardiovascular risk factors was obtained through standardized home interviews, physical examination, and blood sampling [21]. Body mass index was calculated as weight (kg)/height (m)^2^, and obesity was defined as a body mass index of > 30 kg/m^2^. Systolic and diastolic blood pressure were measured twice at the right arm using a random-zero sphygmomanometer; the average of the measurements was used. Hypertension was defined as systolic blood pressure ≥ 140 mmHg and/or diastolic blood pressure ≥ 90 mmHg and/or use of blood pressure lowering medication. Serum total cholesterol and high-density lipoprotein (HDL) cholesterol were assessed using an automatic enzymatic procedure (Hitachi 911, Roche CHOD PAP). Hypercholesterolemia was defined as a serum total cholesterol of ≥ 6.2 mmol/l and/or use of lipid lowering medication [22]. We defined low HDL-cholesterol as < 1.0 mmol/l [22]. Information on antidiabetic medication, blood pressure– and lipid-lowering medication use, and smoking behavior was obtained by trained interviewers. Diabetes mellitus was defined as use of antidiabetic medication, fasting serum glucose level ≥ 7.1 mmol/l, or random serum glucose level ≥ 11.1 mmol/l [23]. Smoking behavior was classified into “current,” and “non-smoking.” We defined history of cardiovascular disease as myocardial infarction, stroke, percutaneous transluminal coronary angioplasty, and/or coronary artery bypass graft prior to baseline examination. Information on myocardial infarction, stroke, percutaneous transluminal coronary angioplasty, and coronary artery bypass graft was obtained through continuous data linkage and monitoring as described previously [24–26].

### Statistical analysis

Baseline characteristics of the study population were provided for the total population, and stratified by sex. Baseline characteristics of the participants that were lost to follow-up (who had complete information on calcification at baseline but did not undergo a follow-up CT scan) were also provided.

For each artery, the incidence of calcification was determined. Incident calcification was defined as detectable calcification at the follow-up examination among participants without calcification at baseline. Next, we assessed the (median) baseline and follow-up volumes of arterial calcification. We calculated absolute change in calcification volumes per artery by subtracting the baseline volume from the follow-up calcification volume (calcification volume at follow-up—baseline calcification volume). Subsequently, to provide insights into the change in calcification relative to the baseline volumes, we calculated relative change in calcification per artery for each person using the following formula: [(calcification volume at follow-up − baseline calcification volume) / baseline calcification volume] × 100%. Annual relative change in arterial calcification was calculated by dividing the relative change volumes by follow-up period. We provided median volumes with interquartile ranges of the overall and annualized relative change in each artery. In addition, per artery, we calculated the proportion of participants who showed regression defined as negative change in volumes of calcification (change < 0 mm^3^) defined as negative change in volumes of calcification (change < 0 mm^3^).

Using Spearman’s rank correlation, we examined the correlation of baseline calcification volume with follow-up calcification volume for each site. We also examined the Spearman’s correlation of calcification volumes across the different arteries (both baseline volumes and relative change volumes). Then, we computed sex-specific quartiles of relative change in calcification volumes, and the upper quartile was defined as “severe progression.” To investigate simultaneous presence of severe progression in different arteries, we assessed all possible combinations of severe progression of CAC, AAC, ECAC, and ICAC. For this analysis, VBAC was omitted because its low prevalence precluded the possibility to create quartiles.

We assessed the association between cardiovascular risk factors with change in calcifications using the following approach. First, considering the skewed distribution, we performed a cube root transformation of the absolute change in calcification [absolute change volume (Δ)]^(^1/3^)]. Second, using linear regression models, we assessed the association of baseline cardiovascular risk factors including age (per 10 years), obesity, hypertension, diabetes, hypercholesterolemia, HDL < 1 mmol/l, current smoking, and history of CVD with the cube root transformed absolute change in calcification volumes. Model 1 included all cardiovascular risk factors, cohort, and follow-up time. Model 2 additionally included the baseline calcification volume. Third, we similarly performed a cube root transformation of the relative change volumes and assessed the association of the abovementioned cardiovascular risk factors with the cube root transformed relative change volumes. We adjusted these analyses for cardiovascular risk factors, cohort, and follow-up time. All analyses were stratified by sex.

To account for missing data of covariables (maximum amount of missingness was 4.2%), we used multiple imputation by chained equations [27]. Analyses were performed using Stata, R (R Foundation for Statistical Computing. http://www.R-project.org/) and RStudio 3.4.4 (http://www.rstudio.org/).

## Results

Baseline characteristics of the study population are shown in Table 1. The mean age of the 807 participants was 65.8 years (standard deviation, ± 4.2) and 54.0% were women. VBAC was least prevalent (men, 12.1%; women, 12.8%) and AAC was most prevalent (men, 89.5%; women, 86.9%). The prevalence of CAC and ECAC was higher in men than in women (CAC in men, 86.3%; in women, 66.1%; ECAC in men, 72.8%; in women, 58.0%). Supplemental Table I shows characteristics of participants who were lost to follow-up. Compared to the population with available follow-up data, participants who were lost to follow-up were older (mean age [± standard deviation], 71.5 years [± 7.0]), and had a higher prevalence of calcification. Particularly participants with VBAC at baseline were often lost to follow-up.

**Table 1:** Baseline characteristics of the study population

Characteristics	Total	Men	Women	*p* value^a^
Number	807	371	436	
Age (years)	65.8 (4.2)	65.8 (3.9)	65.8 (4.4)	0.90
Body mass index (kg/m^2^)	27.6 (3.7)	27.6 (3.4)	27.6 (3.8)	0.76
Systolic blood pressure (mmHg)	142.5 (17.5)	141.9 (17.5)	142.9 (17.5)	0.39
Diastolic blood pressure (mmHg)	81.2 (10.0)	82.9 (9.9)	79.8 (10.0)	< 0.001
Cholesterol in serum (mmol/l)	5.8 (0.9)	5.6 (0.9)	6.0 (0.9)	< 0.001
Glucose in serum (mmol/l)	5.6 (1.3)	5.7 (1.1)	5.6 (1.4)	0.26
HDL-cholesterol in serum (mmol/l)	1.5 (0.4)	1.3 (0.3)	1.6 (0.4)	< 0.001
Hypercholesterolemia, *N* (%)	323 (40.0)	122 (32.9)	201 (46.1)	< 0.001
Diabetes, *N* (%)	78 (10.1)	43 (12.0)	35 (8.5)	0.10
Hypertension, *N* (%)	517 (64.1)	235 (63.5)	282 (64.7)	0.73
Lipid lowering medication, *N* (%)	165 (20.7)	72 (19.8)	93 (21.5)	0.54
Antidiabetic therapy, *N* (%)	40 (5.0)	19 (5.1)	21 (4.9)	0.82
Blood pressure lowering medication, *N* (%)	238 (29.9)	119 (32.7)	119 (27.5)	0.11
Current smokers, *N* (%)	100 (12.7)	58 (16.1)	42 (9.8)	< 0.001
History of cardiovascular disease, *N* (%)	40 ( 5.0)	28 (7.5)	12 (2.8)	0.00
Coronary artery calcification, *N* (%)	608 (75.3)	320 (86.3)	288 (66.1)	< 0.001
Aortic arch calcification, *N* (%)	711 (88.1)	332 (89.5)	379 (86.9)	0.26
Extracranial carotid artery calcification, *N* (%)	523 (64.8)	270 (72.8)	253 (58.0)	< 0.001
Intracranial carotid artery calcification, *N* (%)	600 (74.3)	273 (73.6)	327 (75.0)	0.65
Vertebrobasilar artery calcification, *N* (%)	101 (12.5)	45 (12.1)	56 (12.8)	0.76

Characteristics based on non-imputed data. Provided is the mean (standard deviation) or absolute number (percentage)

^a^*p* value for differences in characteristics between men and women estimated using *t*-test for continuous variables, and chi-square test for categorical variables

### Change in arterial calcification

The graphic abstract provides an example of arterial calcification progression in the different arteries. Among men (*N* = 371), 13.7% had no detectable CAC at baseline. For AAC, this was 10.5%, for ECAC 27.2%, for ICAC 26.4%, and for VBAC 87.9%. Among women (*N* = 436), 33.9% had no detectable CAC at baseline. For AAC, this was 13.1%, for ECAC 42.0%, for ICAC 25.0%, and for VBAC 87.2%. In total, 6 men (1.6%) and 7 women (1.6%) were completely free of calcification at baseline. Among men, 11.6% developed incident CAC, 10.0% incident AAC, 20.8% incident ECAC, 18.9% incident ICAC, and the incidence of VBAC was 31.5%. Among women 24.8% developed incident CAC, 12.4% incident AAC, 29.6% incident ECAC, 19.7% incident ICAC, and the incidence of VBAC was 20.2%. At follow-up, we observed larger median calcification volumes than at baseline in all arteries, except for VBAC. The difference in AAC was most substantial; the median volume (mm^3^) increased from of 129 to 916 in men and from 93 to 839 in women (Table 2).

**Table 2:** Calcification volumes at baseline and follow-up measurements

		Baseline			Follow-up		
		25th percentile	50th percentile	75th percentile	25th percentile	50th percentile	75th percentile
	CAC, mm^3^	5.5	56.9	273.1	131.5	411.1	1102.7
Men,	AAC, mm^3^	19.6	128.5	401.0	320.6	915.8	2190.6
*N* = 371	ECAC, mm^3^	0.0	14.5	73.7	44.4	164.0	398.9
	ICAC, mm^3^	0.0	23.7	85.8	29.6	119.4	324.3
	VBAC, mm^3^	0.0	0.0	0.0	0.0	0.0	9.7
	CAC, mm^3^	0.0	3.3	44.9	14.1	94.6	343.6
Women,	AAC, mm^3^	13.2	92.7	336.3	308.3	838.8	2064.2
*N* = 436	ECAC, mm^3^	0.0	1.3	26.1	11.0	80.6	220.9
	ICAC, mm^3^	0.0	15.8	58.6	25.5	86.9	231.6
	VBAC, mm^3^	0.0	0.0	0.0	0.0	0.0	2.0

*CAC* coronary artery calcification, *AAC* aortic arch calcification, *ECAC* extracranial carotid artery calcification, *ICAC* intracranial carotid artery calcification, *VBAC* vertebrobasilar artery calcification

**Fig. 1:**
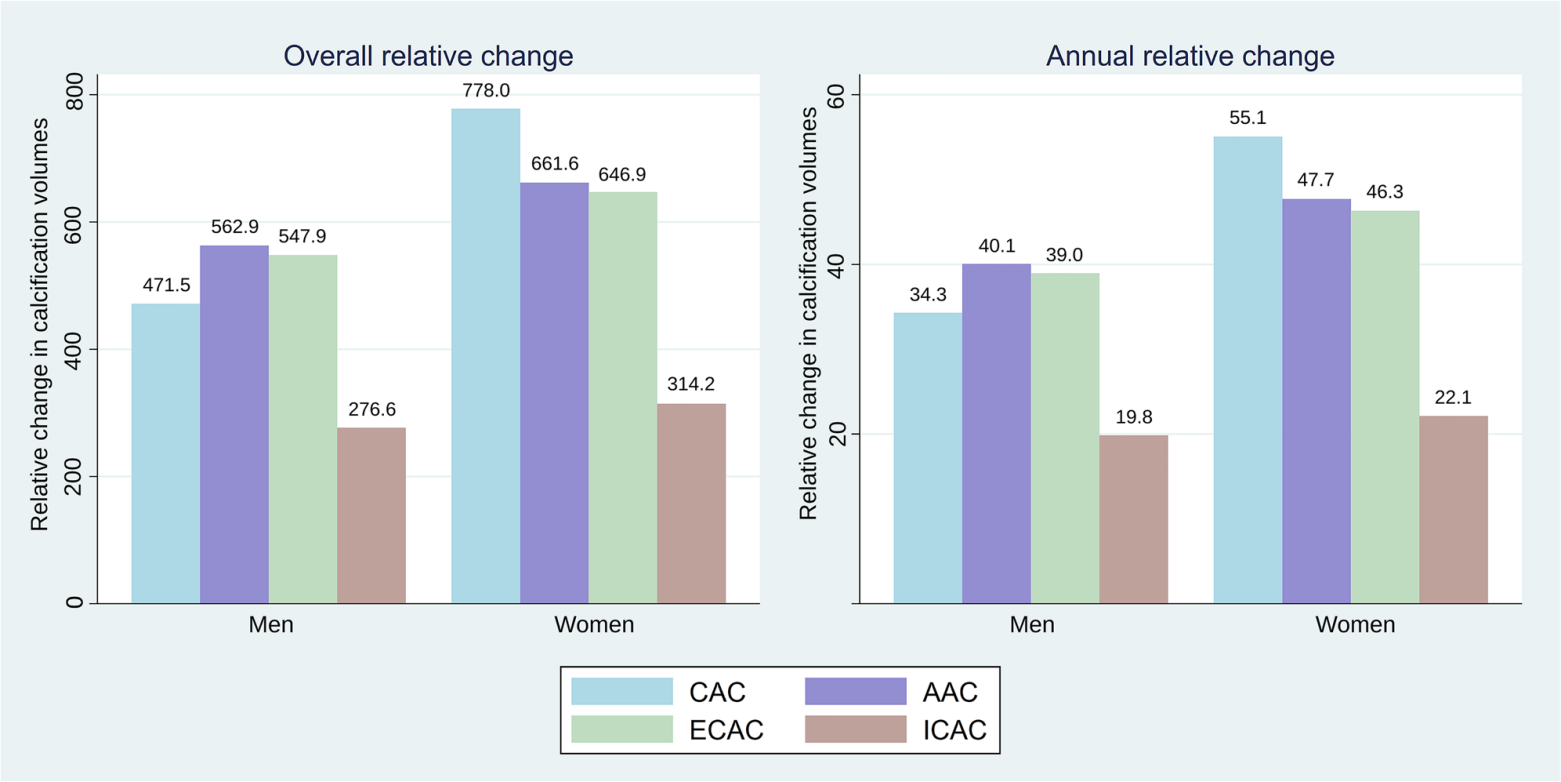
Relative change in calcification volumes. Presented is the median relative change in calcification volumes ([(calcification volume at follow-up − baseline calcification volume) / baseline calcification volume] × 100%). CAC, coronary artery calcification; AAC, aortic arch calcification; ECAC, extracranial carotid artery calcification; ICAC, intracranial carotid artery calcification. Vertebrobasilar artery calcification is precluded because of median relative change of zero

The median relative changes in arterial calcifications are shown in Fig. 1. In men, the median relative change ranged from 278.3% for ICAC to 555.0% for AAC. In women, it ranged from 308.0% for ICAC to 778.0% for CAC. The median annual relative change ranged from 19.9 to 39.2% in men and from 21.9 to 55.1% in women (Fig. 1). Supplemental Table II shows relative changes including interquartile ranges.

Among men, 3.0% showed regression of CAC, 3.5% regression of AAC, 0.8% of ECAC, 5.7% of ICAC, and 1.9% showed regression of VBAC. Among women, 5.2% showed regression of CAC, 1.4% regression of AAC, 1.6% of ECAC, 4.8% of ICAC, and 2.3% showed regression of VBAC.

**Fig. 2:**
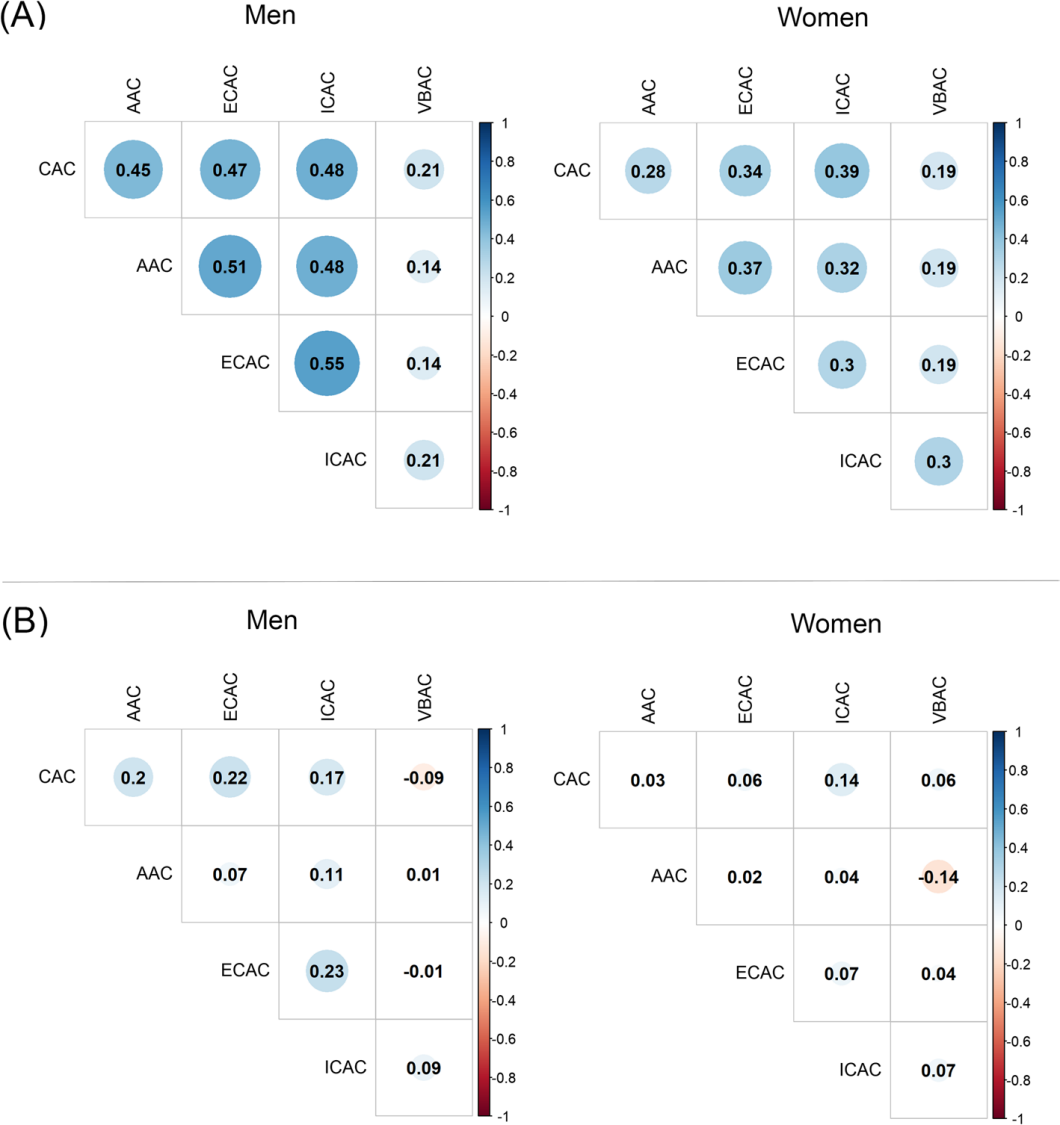
Correlation between baseline calcification volumes and between change in calcification volumes in different arteries. **A** Spearman’s correlation coefficients of the baseline calcification volumes between different arteries. **B** Spearman’s correlation coefficients of the relative change in calcification volumes between different arteries. CAC, coronary artery calcification; AAC, aortic arch calcification; ECAC, extracranial carotid artery calcification; ICAC, intracranial carotid artery calcification; VBAC, vertebrobasilar artery calcification

Among men, the correlation coefficients between calcification at baseline and follow-up were 0.91 for CAC, 0.83 for AAC, 0.85 for ECAC, 0.82 for ICAC, and 0.48 for VBAC (all *p* values < 0.001). Among women, the correlation coefficients were 0.73 for CAC, 0.81 for AAC, 0.76 for ECAC, 0.78 for ICAC, and 0.53 for VBAC (all *p* values < 0.001). The correlation coefficients of relative change in calcification volumes across different arteries ranged from − 0.09 to 0.23 in men and from − 0.14 to 0.14 in women. At baseline, the correlation coefficients of arterial calcification volumes across different arteries ranged from 0.14 to 0.55 in men, from 0.19 to 0.39 in women (Fig. 2).

Supplemental Fig. II shows combinations of relative severe progression of calcification in different arteries. In both men and women, relative severe progression was most often observed in only one artery whereas a combination of relative severe progression in all arteries was least common.

### Cardiovascular risk factors for increase in arterial calcification

**Fig. 3:**
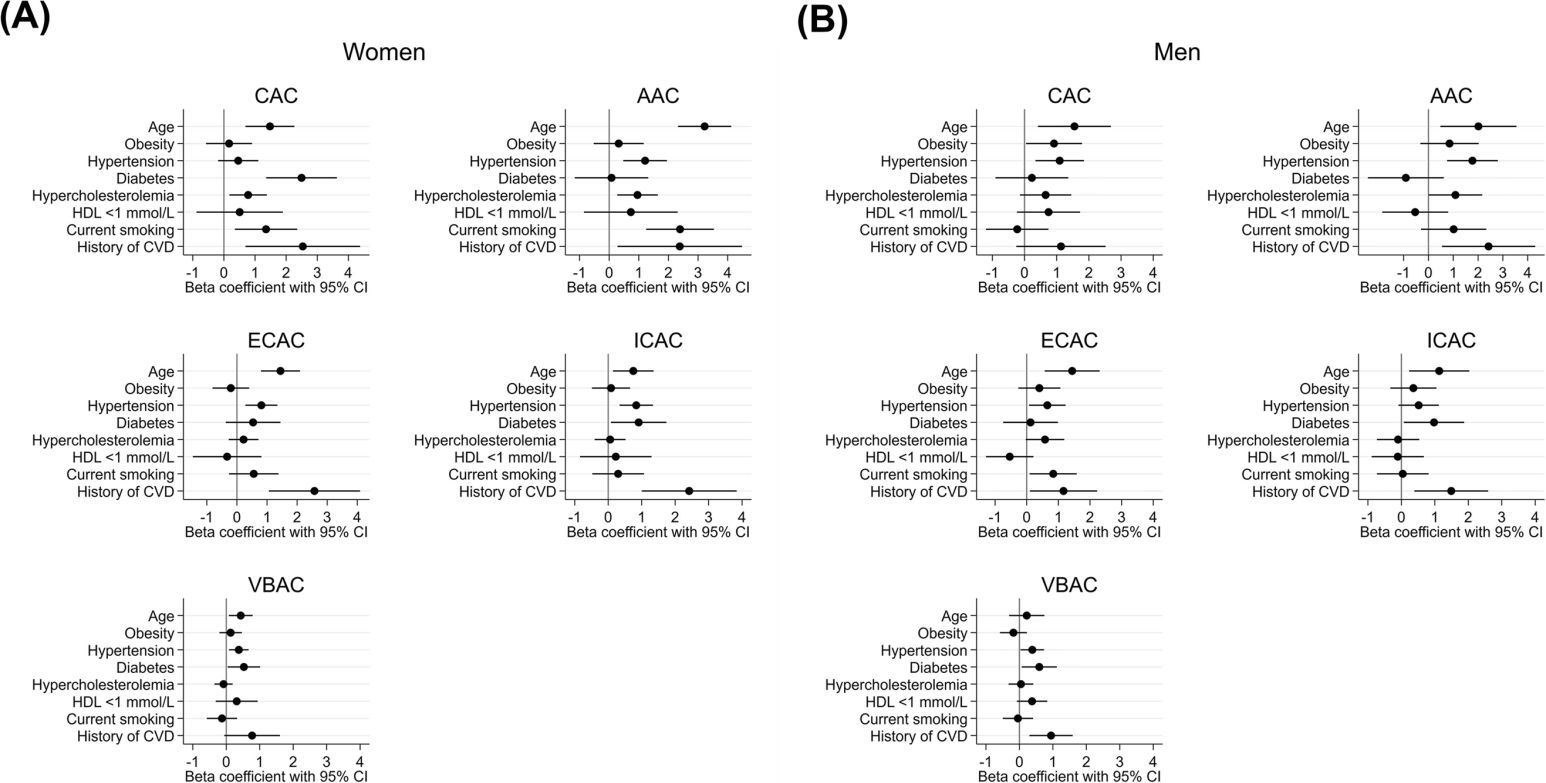
**A** Association between cardiovascular risk factors and absolute change in arterial calcification volumes among women. The estimates represent cube root transformed absolute change in calcification volumes and corresponding 95% confidence intervals per unit increase in age or per cardiovascular risk factor. Associations are adjusted for cohort, all cardiovascular risk factors, and follow-up time. Age represents 10 years of age. CAC, coronary artery calcification; AAC, aortic arch calcification; ECAC, extracranial carotid artery calcification; ICAC, intracranial carotid artery calcification; HDL, high-density lipoprotein; CVD, cardiovascular disease. **B** Association between cardiovascular risk factors and absolute change in arterial calcification volumes among men. The estimates represent cube root transformed absolute change in calcification volumes and corresponding 95% confidence intervals per unit increase in age or per cardiovascular risk factor. Associations are adjusted for cohort, all cardiovascular risk factors, and follow-up time. Age represents 10 years of age. CAC, coronary artery calcification; AAC, aortic arch calcification; ECAC, extracranial carotid artery calcification; ICAC, intracranial carotid artery calcification; HDL, high-density lipoprotein; CVD, cardiovascular disease

Figure 3 shows associations of cardiovascular risk factors with absolute change in arterial calcification per artery in women (Fig. 3A) and in men (Fig. 3B). Hypertension was associated with increases in calcification in all arteries, though the association of hypertension with increase in ICAC in men and with increase in CAC in women did not reach statistical significance. In both women and men, diabetes was associated with increases in ICAC and VBAC. In addition, diabetes was strongly associated with CAC progression in women, whereas in men it was not. In women, smoking was associated with increases in CAC and AAC volumes, whereas in men, smoking was only associated with increase in ECAC volumes. Hypercholesterolemia was associated with CAC and AAC progression in women, and with AAC and ECAC progression in men. Obesity was only associated with CAC progression in men. We found no associations of low HDL-cholesterol with increases in arterial calcification. For beta coefficients and 95% CIs, we refer to Supplemental Table III. After additional adjustment for baseline calcification volume, we found that effect estimates attenuated, and several associations became statistically insignificant (Supplemental Fig. III, and Supplemental Table III for the effect estimates).

Supplemental Fig. IV shows the associations of cardiovascular risk factors with relative change in calcifications. Overall, the associations between cardiovascular risk factors and relative progression of arterial calcification were less prominent, compared to associations with absolute progression. In men and women, the association of diabetes with ICAC and VBAC remained, although only borderline statistically significant. Hypertension was most strongly associated with larger relative increase in ICAC among women. For the beta coefficients and 95% CIs regarding relative change in arterial calcification, we refer to Supplemental Table IV.

## Discussion

The aim of this study was to determine sex-specific changes in arterial calcification in different arteries, and to investigate the influence of cardiovascular risk factors hereon. We found a considerable increase in volumes of arterial calcification over a 14-year time interval. Only for VBAC on average no increase in volumes was observed, though one-quarter of individuals without VBAC at baseline developed VBAC during follow-up. Hypertension was most consistently associated with increases in arterial calcifications. Associations of diabetes, hypercholesterolemia, and smoking with progression of calcifications varied across arteries and sex.

The absolute and relative change in CAC, AAC, ECAC, and ICAC was considerable. While men had larger absolute change volumes, the relative change volumes were larger in women. This observation is largely driven by baseline volumes, which were smaller in women than in men. No data have been published on head-to-head comparisons of changes in arteriosclerosis in different arteries covering an interscan period of more than a decade. However, previous studies demonstrated that the correlation of arterial calcification—measured at a single time-point—is only weak to moderate across different arteries [6, 28]. Our findings extend current knowledge by showing a weak correlation of change in arterial calcification across different arteries. Compared to correlations between calcification volumes at baseline, the correlation of change in arterial calcification across arteries was weaker. In women, correlations of the baseline and the change volumes were weaker than those in men. In addition, we found that severe progression of arterial calcification was often present in only one artery within an individual. This indicates that not only the life-time accumulation of calcification—as captured in one single measure—differs across arteries, but also changes itself. This also suggests that when assessing changes in only one artery, this may not provide a good reflection of the systemic development of arteriosclerosis, even more so in women. Explanations for the artery-specific differences may partly be genetic susceptibility [29], and differences in anatomy and turbulent flow across different segments of the vasculature.

In our study, depending on the artery, up to 6% of the participants showed regression of arterial calcification. This shows that the development of arteriosclerosis is dynamic even on the long-term. Yet it has to be mentioned that regression might partly have signified stabilization as it is challenging to distinguish a minor negative change from potential measurement error. Therefore, these findings should be interpreted with caution.

It is still rather inconclusive whether and how cardiovascular risk factors influence the course of arteriosclerosis. Previously, the Multi-ethnic Study of Atherosclerosis (MESA) has reported on 10-year CAC progression and found that several risk factors were associated with progression of CAC including age, hypertension, and diabetes. This is roughly in line with our findings though we found that diabetes was associated with CAC change in women only whereas hypertension was associated with CAC change in men only. In addition, the Tromsø Study has reported that total cholesterol, blood pressure, and smoking are predictors for 13-year progression of carotid arteriosclerosis, which is comparable to our findings for ECAC among men. In our study, overall, hypertension was most consistently associated with changes in arterial calcifications across arteries and sex. It was noticeable that, in both women and men, diabetes was associated with ICAC and VBAC. This is in line with previous studies showing that diabetes is an independent risk factor for intracranial arteriosclerosis [30, 31]. Furthermore, associations of hypercholesterolemia and smoking varied depending on the artery and sex.

Most previous population-based studies that assessed changes in calcification focused solely on the coronary arteries. Besides MESA [9, 32], few other population-based studies have shown associations of cardiovascular risk factors, including high cholesterol and high fasting insulin levels, with CAC progression [33, 34]. Yet, CAC progression has also been described as an inevitable process with minimal influence of cardiovascular risk factors [35]. The discrepancies in literature could partly be explained by different methods to define change or progression. For example, in their definition of progression, some studies have included all study participants whereas others have solely included participants with a baseline value of > 0, and thus excluded persons without calcification at baseline. This makes it challenging to compare our results with other studies. In addition, it matters whether absolute change or relative change is used as outcome variable. We found that the strength of the associations between cardiovascular risk factors and absolute change in calcification slightly attenuated after adjustment for baseline calcification volume. We further endeavored to rule out the effect of lifetime accumulation of arterial calcification by using relative change as outcome measure. However, this measure is highly influenced by small baseline volumes. Indeed, associations were less prominent when we took relative change instead of absolute change as an outcome. As such, the burden of arteriosclerosis as measured by a single, one-time assessment of arteriosclerosis may provide a better reflection of the vascular disease burden than changes in arteriosclerosis.

The unique study setting allowed to investigate artery-specific changes in arteriosclerosis over a long time period in a population-based sample. Besides its uniqueness, an advantage of the long timespan is that the impact of systemic measurement error is limited as large changes are likely to be captured whereas small changes on the short-term might be more easily overlooked or too small to measure. However, we also need to address several methodological considerations. Some potentially important associations between risk factors and changes in calcification might not have reached statistical significance due to limited power. Furthermore, survival bias and non-response bias may have led to an underestimation of the changes in calcification and the influence of cardiovascular risk factors on these changes as the unhealthiest individuals are most likely to have died and be among the non-responders, whereas healthier individuals often volunteer to undergo a follow-up examination. Indeed, we found that participants who underwent a follow-up examination were younger and had a more favorable cardiovascular risk profile compared to those who did not undergo a second CT scan. This also partly explains why we did not find pronounced changes in VBAC volumes over time and—in men—we did not find associations between age and VBAC even though VBAC is highly age-related [17]. Another methodological issue to consider is that our study population is almost exclusively limited to persons from European descent. This means that our findings are not plainly generalizable to other parts of the globe. In addition, a limitation of our study is that it includes only two CT examinations. Availability of one or more scans in between would have provided additional insights into the course of arteriosclerosis over time, including its dynamic nature [5]. Moreover, along with the advances in imaging techniques, the scanner used at follow-up was a different type than the scanner used at baseline. Yet both scanners were from the same vendor and scan parameters were kept similar. Nonetheless, we cannot rule out potential differences in calcium estimations due to inter-scanner variability. However, given the long interscan period along with considerable changes in calcification, potential small measurement errors would not change the overall interpretation of our results.

The findings of our study provide insights into the course of arteriosclerosis, and may help to further explain the weak to moderate correlation of arteriosclerosis across arteries. Still, the utility of assessing changes in arteriosclerosis is not clear yet. In particular, when measuring change in arterial calcification, it is challenging to distinguish between threatening deterioration of vascular health on one hand and stabilization of plaque on the other hand [36]. Moreover, associations between cardiovascular risk factors and changes in arteriosclerosis over time may still partly be a reflection of the life-time accumulation of arteriosclerosis instead of the progression or change over the 14 years we endeavored to capture. To overcome all these challenges, future research should include multiple examinations of arteriosclerosis, focus on specific components of arteriosclerosis beyond arterial calcification, and further assess the value of serial assessments of arteriosclerosis for the prediction of cardiovascular disease. In our study, using non-contrast CT, we were unable to visualize the branches of the vessels. For future studies, assessment of proximity between vessel branches and the presence of calcifications could provide additional insights into anatomical differences underlying differences in the development of arteriosclerosis.

In conclusion, in this population-based cohort covering a 14-year timespan, we found considerable progression of arterial calcification in different arteries. On individual level, severe progression was often observed in only one artery, suggestive of artery-specific differences in the rate of change in arterial calcification over time. Hypertension was most consistently associated with increase in arterial calcifications. Associations of diabetes, hypercholesterolemia, and smoking with increases in calcification varied across arteries and sex. Though cardiovascular risk factors were associated with changes in arterial calcifications, the observed associations may still partly be driven by the lifetime accumulation of arterial calcification.

### Supplementary Information

Below is the link to the electronic supplementary material.

Supplementary file1 (PDF 343 KB)

## Abbreviations

AAC: Aortic arch calcification
CAC: Coronary artery calcification
ECAC: Extracranial carotid artery calcification
ICAC: Intracranial carotid artery calcification
VBAC: Vertebrobasilar artery calcification

## Declarations

### Guarantor

The scientific guarantor of this publication is Daniel Bos.

### Statistics and biometry

No complex statistical methods were necessary.

### Study subjects or cohorts overlap

The current study includes a population who underwent CT examinations within the Rotterdam Study. Based on the cohort, several studies have been published covering different research questions.

### Methodology

• prospective

• observational

• multicentre study
