# Supplementary material for: Progression of arterial calcifications: what, where, and in whom?
Source: Eur Radiol. 2024 Jan 15;34(8):5142–52. doi: 10.1007/s00330-023-10566-7 (PMC11254972; doi:10.1007/s00330-023-10566-7)
Supplement: Supplementary file 1 — Supplementary file1 (PDF 343 KB) [file 330_2023_10566_MOESM1_ESM.pdf]

Progression of arterial calcifications: what, where, and in whom?

Electronic Supplementary Material

Methods	- Scan protocol
Tables	-Table I. Baseline characteristics of the study population and of the non-participants
	-Table II. Relative change in calcification volumes
	-Table III. Association between cardiovascular risk factors and absolute change in arterial calcification
	-Table IV. Association between cardiovascular risk factors and relative change in arterial calcification
Figures	-Fig I. Flowchart of the study population
	-Fig. II. Combinations of relative severe progression of calcification in different arteries
	-Fig. III. Association between cardiovascular risk factors and cube root transformed - absolute change in arterial calcification volumes
	-Fig. IV. Association between cardiovascular risk factors and cube root transformed relative change in arterial calcification volumes

Eur Radiol (2023) van der Toorn JE, Vernooij MW, Ikram MA, Kavousi M, Bos D.

Scan protocol

At baseline, non-contrast CT images were obtained using 16-slice or 64-slice *(z-flying focal spot (z-FFS))* MDCT scanners (SOMATOM Sensation 16 or 64; Siemens). Two scans were performed: a cardiac and a carotid scan. The cardiac scan covered the apex of the heart to the tracheal bifurcation. The carotid scan ranged from the aortic root to the Circle of Willis (1cm above the sella turcica). With the 16-slice scanner, cardiac images were obtained within a single breath-hold, with the following parameters: 12 mm × 1.5 mm collimation, 120 kVp, effective 30 mAs, prospective ECG triggering at 50% of the cardiac cycle. The scan parameters of the carotid scan were: 16 × 0.75 mm collimation, 120 kVp, 100 effective mAs, 0.5 s rotation time and normalized pitch of 1. For the 64-slice scanner, parameters were similar, except for collimation and effective mAs. The collimation was 32 × 0.6 mm and the mAs value was real time adapted to body weight (CARE Dose 4D, Siemens).

During the follow-up examination, non-contrast CT images were obtained using a 128-slice (z-FFS) dual source CT (DSCT) scanner (SOMATOM Drive, Siemens). Similar to the baseline examination, a cardiac and carotid scan were performed. Within a single breath-hold cardiac images were acquired with 64 mm × 0.6 mm collimation, 120 kVp, and effective 80 mAs (CARE Dose4D). The scan parameters of the carotid scan were: 64 × 0.6 mm collimation, 120 kVp, 50 effective mAs (CARE Dose4D), 0.5 s rotation time and normalized pitch of 0.8.

At baseline and follow-up, the matrix size was 512-by-512, and acquired images were reconstructed with an optimized field-of-view of 180 and 120 for the cardiac and carotid scans, respectively, slice thickness of 3.0 and 1.0 mm, and an increment of 1,5 and 0.5 mm using a medium non-iterative convolution kernel (B35f). On both baseline and follow-up scans, calcifications were assessed in the coronary arteries, aortic arch, extracranial, and intracranial carotid arteries, and the vertebrobasilar arteries. Coronary artery calcification (CAC), aortic arch calcification (AAC), and extracranial carotid artery calcification (ECAC) were quantified (in mm^3) using dedicated software (Syngo Calcium Scoring; Siemens). Intracranial carotid artery calcification (ICAC) and vertebrobasilar artery calcification (VBAC) were quantified using a semiautomatic scoring method that allows to manually segment calcification in each consecutive CT-slice after which the calcification volume is computed using the pixel size and the increment

Table I. Baseline characteristics of the study population and of the non-participants

Characteristics	Study population	Non-participants	p-value
No.	N=807	N=1,577	
Sex, women	436 (54.0%)	818 (51.9%)	0.32
Age (years)	65.8 (4.2)	71.5 (7.0)	<0.001
Body mass index (kg/m^2)	27.6 (3.7)	27.6 (4.2)	0.99
Systolic blood pressure (mmHg)	142.5 (17.5)	148.9 (21.0)	<0.001
Diastolic blood pressure (mmHg)	81.2 (10.0)	79.7 (11.1)	0.001
Cholesterol in serum (mmol/l)	5.8 (0.9)	5.6 (1.0)	<0.001
Glucose in serum (mmol/l)	5.6 (1.3)	5.8 (1.3)	0.004
HDL-cholesterol in serum (mmol/l)	1.5 (0.4)	1.4 (0.4)	0.012
Diabetes	78 (10.1%)	218 (14.9%)	0.001
Hypercholesterolemia	324 (40.1%)	665 (42.2%)	0.34
Hypertension	517 (64.1%)	1,247 (79.4%)	<0.001
Lipid reducing medication	165 (20.7%)	380 (24.5%)	0.040
Current smokers	100 (12.7%)	267 (17.4%)	0.001
History of cardiovascular disease	40 (5.0%)	198 (12.6%)	<0.001
CAC	608 (75.3%)	1,349 (85.5%)	<0.001
AAC	711 (88.1%)	1,499 (95.1%)	<0.001
ECAC	523 (64.8%)	1,222 (77.5%)	<0.001
ICAC	600 (74.3%)	1,351 (85.7%)	<0.001
VBAC	101 (12.5%)	385 (24.4%)	<0.001

Comparison of baseline characteristics of the current study population versus participants who had complete information of calcification at baseline but did not undergo a follow-up examination. Values are based on non-imputed data.

[a]P-value for differences in characteristics between the groups estimated using t-test or Mann-Whitney U test for (skewed) continuous variables, and chi-square test for categorical variables. CAC, coronary artery calcification; AAC, aortic arch calcification; ECAC, extracranial carotid artery calcification; ICAC, intracranial carotid artery calcification; VBAC, vertebrobasilar artery calcification.

Table II. Relative change in calcification volumes

		Percentage change in calcification			Annual percentage change in calcification		
		25th percentile	50th percentile	75th percentile	25th percentile	50th percentile	75th percentile
Men, N=371	CAC, mm^3	205.1	471.5	1305.8	14.4	34.3	92.3
	AAC, mm^3	251.8	558.3	1894.6	17.8	39.5	135.6
	ECAC, mm^3	213.1	547.9	1860.0	15.3	39.0	132.7
	ICAC, mm^3	111.6	278.3	818.8	7.8	19.9	57.2
	VBAC, mm^3	0.0	0.0	443.5	0.0	0.0	31.5
Women, N=436	CAC, mm^3	198.4	778.0	3681.6	14.0	55.0	259.8
	AAC, mm^3	321.2	661.7	2175.7	22.3	47.7	151.4
	ECAC, mm^3	163.6	646.9	2806.1	11.7	46.3	200.1
	ICAC, mm^3	105.6	314.2	926.5	7.6	22.1	65.7
	VBAC, mm^3	0.0	0.0	98.7	0.0	0.0	6.9

Values represent relative change in calcification volumes ([(calcification volume at follow-up – baseline calcification volume) / baseline calcification volume] * 100%). CAC, coronary artery calcification; AAC, aortic arch calcification; ECAC, extracranial carotid artery calcification; ICAC, intracranial carotid artery calcification; VBAC, vertebrobasilar artery calcification.

Table III. Association between cardiovascular risk factors and absolute change in arterial calcification

			CAC		AAC		ECAC		ICAC		VBAC	
		**Model**	**β**	**95%-CI**	**β**	**95%-CI**	**β**	**95%-CI**	**β**	**95%-CI**	**β**	**95%-CI**
	Age	1	1.55	0.42 - 2.69	2.01	0.48 - 3.55	1.44	0.57 - 2.30	1.13	0.23 - 2.03	0.22	-0.31 - 0.75
		2	1.46	0.33 - 2.59	0.75	-0.57 - 2.07	0.70	-0.03 - 1.43	0.31	-0.51 - 1.13	0.30	-0.19 - 0.80
	Obesity	1	0.92	0.05 - 1.79	0.85	-0.33 - 2.03	0.40	-0.27 - 1.07	0.36	-0.33 - 1.05	-0.18	-0.58 - 0.23
		2	0.81	-0.05 - 1.68	0.40	-0.60 - 1.40	-0.10	-0.66 - 0.46	0.23	-0.38 - 0.85	-0.19	-0.57 - 0.18
Men	Hypertension	1	1.09	0.34 - 1.85	1.77	0.75 - 2.80	0.65	0.07 - 1.23	0.52	-0.09 - 1.12	0.38	0.03 - 0.74
		2	1.09	0.34 - 1.84	1.07	0.20 - 1.95	0.39	-0.09 - 0.88	0.53	0.00 - 1.07	0.32	-0.01 - 0.65
	Diabetes	1	0.23	-0.90 - 1.36	-0.91	-2.44 - 0.62	0.12	-0.74 - 0.99	0.98	0.07 - 1.88	0.59	0.07 - 1.12
		2	0.27	-0.85 - 1.40	-0.48	-1.78 - 0.82	0.41	-0.31 - 1.12	1.26	0.46 - 2.06	0.42	-0.07 - 0.91
	Hypercholesterolemia	1	0.66	-0.14 - 1.45	1.09	0.01 - 2.17	0.58	-0.03 - 1.19	-0.10	-0.73 - 0.53	0.05	-0.32 - 0.42
		2	0.61	-0.19 - 1.40	0.60	-0.32 - 1.52	0.44	-0.07 - 0.94	-0.25	-0.82 - 0.31	-0.05	-0.40 - 0.29
	HDL<1mmol/L	1	0.75	-0.23 - 1.73	-0.53	-1.86 - 0.80	-0.54	-1.29 - 0.21	-0.11	-0.89 - 0.67	0.38	-0.08 - 0.83
		2	0.82	-0.15 - 1.80	0.22	-0.91 - 1.35	-0.26	-0.89 - 0.36	0.03	-0.66 - 0.73	0.20	-0.23 - 0.63
	Smoking	1	-0.23	-1.20 - 0.75	1.02	-0.30 - 2.33	0.84	0.09 - 1.58	0.04	-0.73 - 0.81	-0.04	-0.50 - 0.41
		2	-0.28	-1.25 - 0.68	0.55	-0.57 - 1.67	0.38	-0.24 - 1.01	-0.07	-0.75 - 0.62	-0.01	-0.43 - 0.41
	History of CVD	1	1.13	-0.25 - 2.52	2.42	0.54 - 4.30	1.16	0.10 - 2.23	1.49	0.39 - 2.59	0.95	0.30 - 1.59
		2	0.61	-0.83 - 2.05	1.82	0.23 - 3.42	0.26	-0.63 - 1.16	0.34	-0.67 - 1.35	0.65	0.05 - 1.26
	Age	1	1.48	0.69 - 2.27	3.22	2.32 - 4.12	1.45	0.80 - 2.10	0.75	0.14 - 1.36	0.43	0.08 - 0.79
		2	1.16	0.42 - 1.91	1.41	0.60 - 2.23	0.93	0.35 - 1.52	-0.06	-0.63 - 0.50	0.33	0.01 - 0.65
	Obesity	1	0.17	-0.57 - 0.91	0.32	-0.52 - 1.17	-0.20	-0.81 - 0.41	0.08	-0.49 - 0.65	0.13	-0.20 - 0.47
		2	0.13	-0.57 - 0.82	0.30	-0.42 - 1.02	-0.10	-0.64 - 0.45	0.07	-0.44 - 0.58	0.19	-0.11 - 0.49
Women	Hypertension	1	0.46	-0.19 - 1.11	1.21	0.47 - 1.95	0.81	0.28 - 1.34	0.84	0.34 - 1.34	0.37	0.08 - 0.67
		2	0.27	-0.34 - 0.88	0.90	0.27 - 1.53	0.66	0.18 - 1.14	0.77	0.32 - 1.21	0.27	0.01 - 0.54
	Diabetes	1	2.50	1.36 - 3.63	0.08	-1.16 - 1.32	0.54	-0.37 - 1.45	0.91	0.08 - 1.74	0.53	0.04 - 1.01
		2	2.25	1.18 - 3.31	-0.04	-1.10 - 1.03	0.15	-0.66 - 0.96	1.06	0.31 - 1.80	0.60	0.16 - 1.03
	Hypercholesterolemia	1	0.78	0.18 - 1.38	0.96	0.27 - 1.64	0.22	-0.27 - 0.71	0.06	-0.41 - 0.52	-0.08	-0.35 - 0.19
		2	0.63	0.06 - 1.20	0.95	0.37 - 1.53	0.17	-0.27 - 0.61	0.04	-0.38 - 0.45	-0.10	-0.35 - 0.14

HDL<1mmol/L	1	0.51	-0.88 - 1.90	0.73	-0.85 - 2.31	-0.33	-1.47 - 0.81	0.23	-0.84 - 1.29	0.31	-0.31 - 0.94
	2	0.76	-0.55 - 2.07	0.87	-0.48 - 2.21	-0.65	-1.67 - 0.37	0.37	-0.60 - 1.33	0.29	-0.27 - 0.86
Smoking	1	1.35	0.35 - 2.36	2.39	1.25 - 3.54	0.56	-0.27 - 1.38	0.30	-0.47 - 1.07	-0.13	-0.58 - 0.32
	2	1.28	0.34 - 2.23	1.78	0.80 - 2.76	0.43	-0.31 - 1.16	0.08	-0.61 - 0.78	-0.03	-0.44 - 0.38
History of CVD	1	2.53	0.69 - 4.38	2.38	0.28 - 4.49	2.58	1.06 - 4.09	2.42	1.00 - 3.84	0.77	-0.06 - 1.60
	2	1.14	-0.64 - 2.91	0.50	-1.31 - 2.31	0.60	-0.81 - 2.00	1.07	-0.23 - 2.37	0.32	-0.44 - 1.07

Model 1 is adjusted for cohort, all cardiovascular risk factors, and follow-up time. Model 2 is additionally adjusted for baseline calcification volume. Age represents 10 years of age. CAC, coronary artery calcification; AAC, aortic arch calcification; ECAC, extracranial carotid artery calcification; ICAC, intracranial carotid artery calcification; VBAC, vertebrobasilar artery calcification; HDL, high-density lipoprotein; CVD, cardiovascular disease.

Table IV. Association between cardiovascular risk factors and relative change in arterial calcification

		CAC		AAC		ECAC		ICAC		VBAC	
		β	95%-CI	β	95%-CI	β	95%-CI	β	95%-CI	β	95%-CI
	Age	-1.46	-3.65 - 0.72	-3.69	-6.82 - -0.56	-0.33	-2.55 - 1.90	-0.82	-2.81 - 1.16	1.28	-0.67 - 3.22
Men	Obesity	-0.33	-2.01 - 1.35	-1.98	-4.38 - 0.42	-1.13	-2.83 - 0.58	-0.15	-1.68 - 1.37	-0.58	-2.07 - 0.91
	Hypertension	-0.51	-1.97 - 0.94	-0.71	-2.79 - 1.37	0.27	-1.21 - 1.75	1.37	0.04 - 2.69	0.60	-0.70 - 1.90
	Diabetes	0.96	-1.20 - 3.13	-1.84	-4.96 - 1.27	1.82	-0.38 - 4.02	1.72	-0.25 - 3.69	2.36	0.41 - 4.30
	Hypercholesterolemia	-0.18	-1.72 - 1.36	-0.36	-2.56 - 1.84	0.06	-1.50 - 1.63	-0.50	-1.90 - 0.90	-0.35	-1.72 - 1.02
	HDL<1mmol/L	0.61	-1.29 - 2.50	2.35	-0.36 - 5.06	-0.07	-2.00 - 1.86	0.61	-1.11 - 2.33	0.43	-1.25 - 2.12
	Smoking	-0.41	-2.29 - 1.47	-0.80	-3.48 - 1.89	-0.42	-2.33 - 1.49	-0.67	-2.38 - 1.03	-0.21	-1.88 - 1.46
	History of CVD	-2.81	-5.49 - -0.14	3.58	-0.26 - 7.41	-1.49	-4.21 - 1.23	0.34	-2.09 - 2.77	3.46	1.08 - 5.84
	Age	0.83	-1.40 - 3.06	-4.42	-7.26 - -1.59	0.75	-1.44 - 2.95	-1.83	-3.34 - -0.32	1.30	-0.05 - 2.65
Women	Obesity	0.04	-2.06 - 2.13	-0.27	-2.94 - 2.39	-0.76	-2.83 - 1.30	-0.12	-1.54 - 1.29	1.30	0.03 - 2.57
	Hypertension	1.93	0.10 - 3.77	-0.14	-2.47 - 2.19	1.85	0.04 - 3.65	2.09	0.85 - 3.33	1.16	0.05 - 2.27
	Diabetes	0.95	-2.13 - 4.03	0.22	-3.68 - 4.11	-0.34	-3.36 - 2.69	1.95	-0.10 - 4.01	2.05	0.21 - 3.90
	Hypercholesterolemia	0.89	-0.81 - 2.58	-2.31	-4.47 - -0.14	0.22	-1.45 - 1.89	-0.34	-1.49 - 0.81	-0.22	-1.25 - 0.81
	HDL<1mmol/L	1.66	-2.27 - 5.59	-0.22	-5.22 - 4.77	-1.33	-5.20 - 2.53	-0.05	-2.71 - 2.61	0.42	-1.97 - 2.80
	Smoking	0.99	-1.85 - 3.83	1.63	-1.99 - 5.24	-0.35	-3.15 - 2.44	-0.19	-2.11 - 1.72	-0.01	-1.73 - 1.71
	History of CVD	-2.63	-7.85 - 2.58	-5.08	-11.72 - 1.56	-0.94	-6.08 - 4.20	0.41	-3.12 - 3.94	0.78	-2.38 - 3.94

Adjusted for cohort, all cardiovascular risk factors, and follow-up time. Age represents 10 years of age. CAC, coronary artery calcification; AAC, aortic arch calcification; ECAC, extracranial carotid artery calcification; ICAC, intracranial carotid artery calcification; VBAC, vertebrobasilar artery calcification; HDL, high-density lipoprotein; CVD, cardiovascular disease.

Fig I. Flowchart of the study population

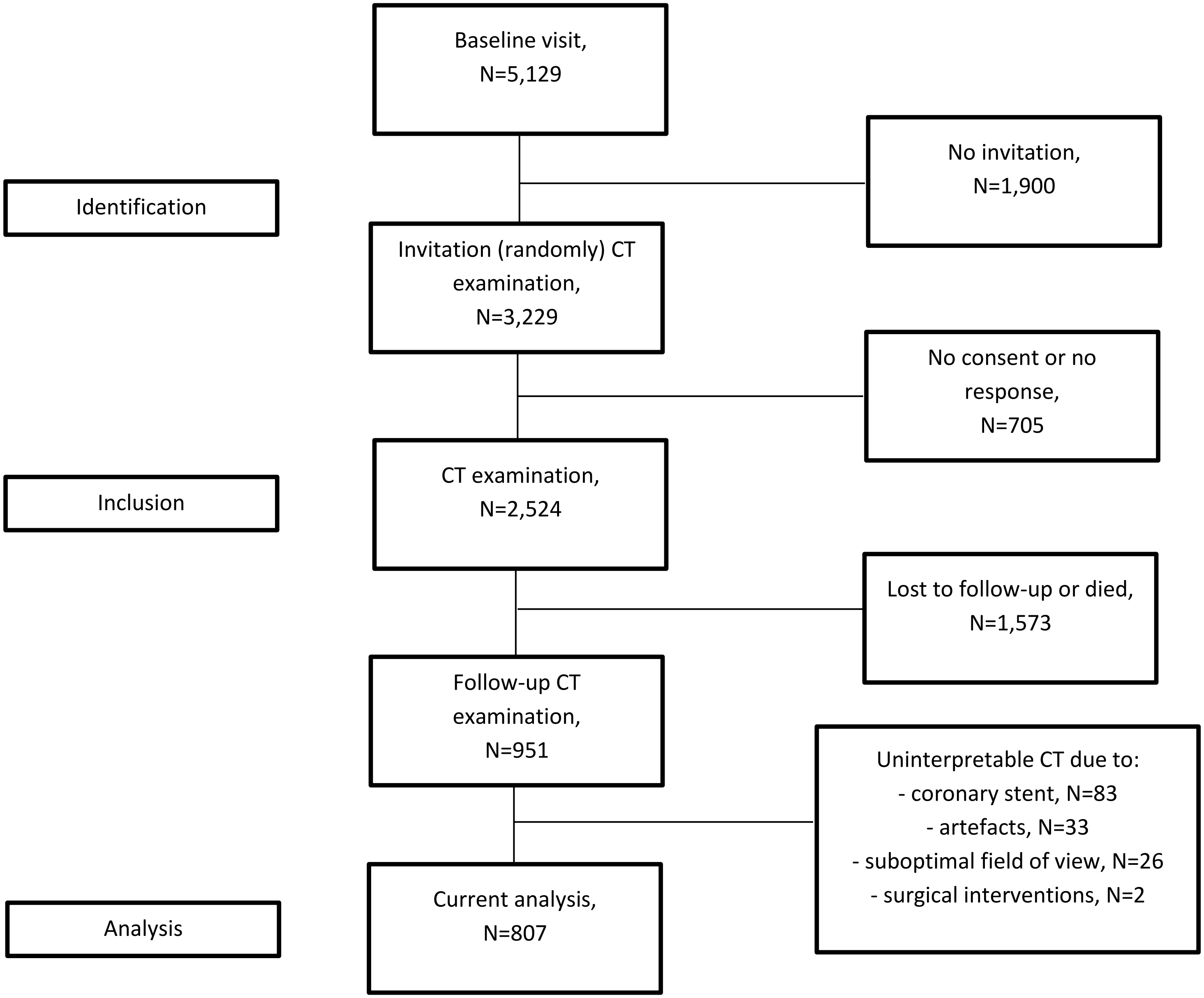

Fig. II. Combinations of relative severe progression of calcification in different arteries

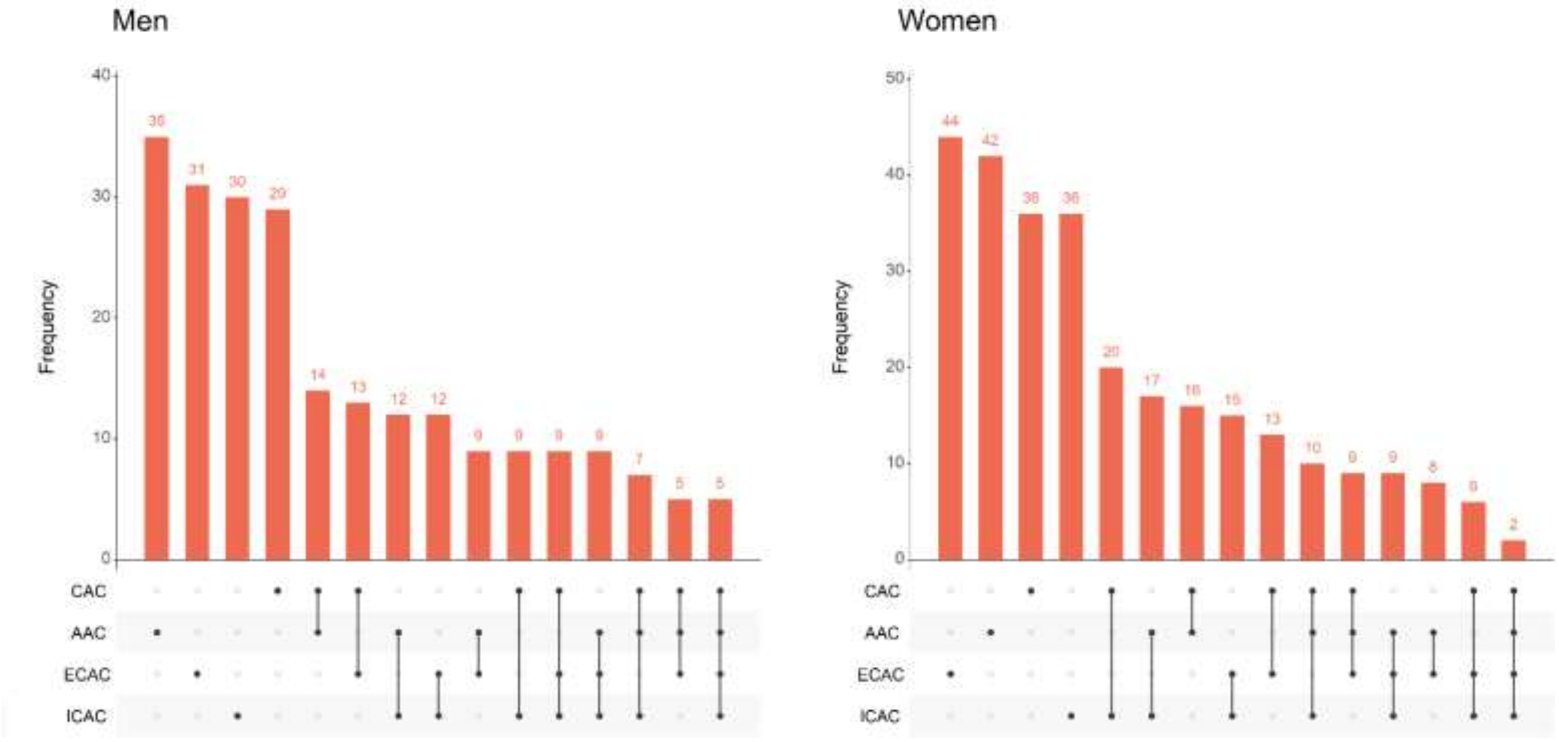

Figure represents combinations of relative severe progression of calcification in different arteries. First, relative change was calculated for each vessel bed as: [(calcification volume at follow-up – baseline calcification volume) / baseline calcification volume] * 100%. Second, severe progression was defined as the upper quartile of relative change, for each vessel bed. CAC, coronary artery calcification; AAC, aortic arch calcification; ECAC, extracranial carotid artery calcification; ICAC, intracranial carotid artery calcification. Note: vertebrobasilar artery calcification is not shown as its low prevalence precluded the possibility to create quartiles.

Fig. III. Association between cardiovascular risk factors and cube root transformed absolute change in arterial calcification volumes

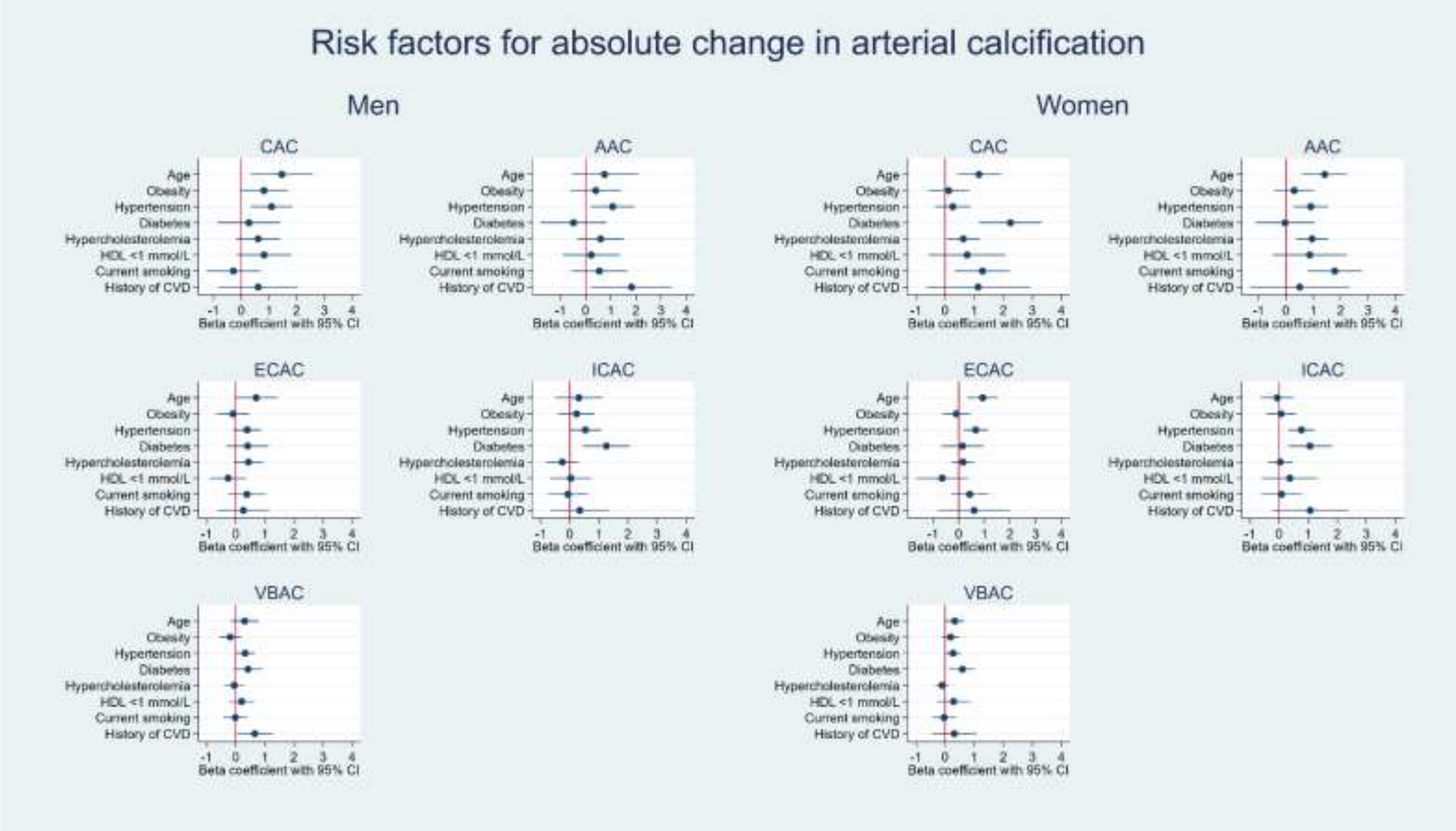

Adjusted for cohort, all cardiovascular risk factors, follow-up time, and baseline volume. Age represents 10 years of age. CAC, coronary artery calcification; AAC, aortic arch calcification; ECAC, extracranial carotid artery calcification; ICAC, intracranial carotid artery calcification; VBAC, vertebrobasilar artery calcification; HDL, high-density lipoprotein; CVD, cardiovascular disease.

Fig. IV. Association between cardiovascular risk factors and cube root transformed relative change in arterial calcification volumes

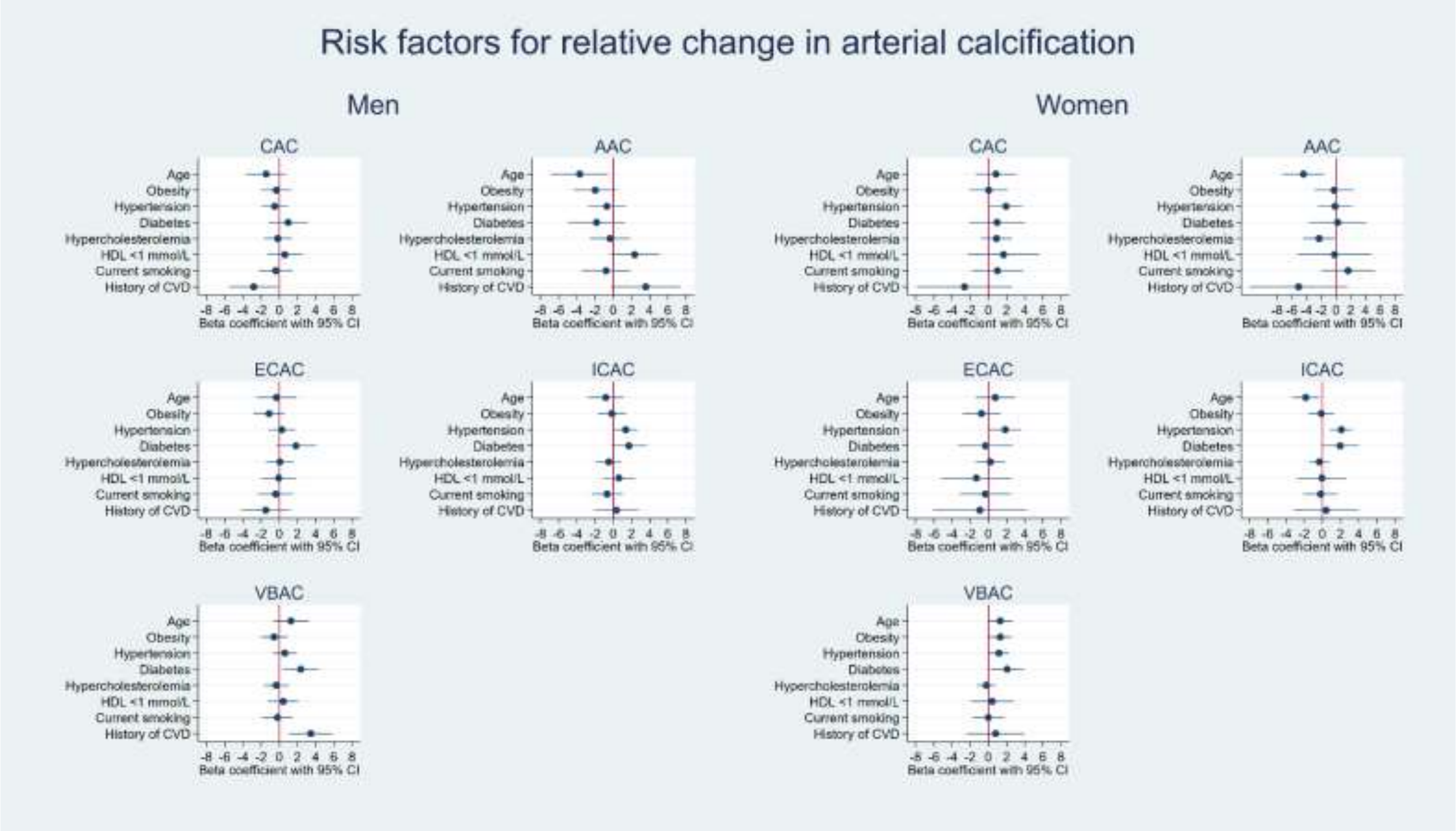

Adjusted for cohort, all cardiovascular risk factors, and follow-up time. Age represents 10 years of age. CAC, coronary artery calcification; AAC, aortic arch calcification; ECAC, extracranial carotid artery calcification; ICAC, intracranial carotid artery calcification; VBAC, vertebrobasilar artery calcification; HDL, high-density lipoprotein; CVD, cardiovascular disease.
